# An Innovative Concept for a Walker with a Self-Locking Mechanism Using a Single Mechanical Approach

**DOI:** 10.3390/ijerph16101671

**Published:** 2019-05-14

**Authors:** Stephane Lopes, Lúcia Filipe, Rosana Silva, Arménio Cruz, Pedro Parreira, Filipa Couto, Rafael Bernardes, João Apóstolo, Luís Roseiro, Cândida Malça

**Affiliations:** 1 Polytechnic Institute of Coimbra, Coimbra Institute of Engineering, 3030-199 Coimbra, Portugal; stephanelopes22@hotmail.com (S.L.); lroseiro@isec.pt (L.R.); 2 Nursing School of Coimbra, 3046-851 Coimbra, Portugal; luciamontenegrofilipe@gmail.com (L.F.); rosanamjsilva82@gmail.com (R.S.); 3 The Health Sciences Research Unit: Nursing, Nursing School of Coimbra, 3046-851 Coimbra, Portugal; acruz@esenfc.pt (A.C.); parreira@esenfc.pt (P.P.); filipadccouto@esenfc.pt (F.C.); rafaelalvesbernardes@esenfc.pt (R.B.); apostolo@esenfc.pt (J.A.)

**Keywords:** technical aids, active aging, walker, self-locking mechanism, self-help devices

## Abstract

*Background:* The ageing process involves a natural degeneration of physiological function and can imply life constraints, namely during activities of daily life (ADL). Walking can be strongly affected by strength, gait, and balance changes, which affect quality of life. The quality of life of the older adult is associated with available solutions that contribute to an active and safe ageing process. Most of these solutions involve technical aids that should be adapted to older adults’ conditions. *Aim*: To identify the advantages and disadvantages of two-wheeled walkers and of two different self-locking systems designed and developed by the authors. *Methods*: Two studies were performed based on the possible walker combinations used, using a walker with no wheels (classic fixed walker), a two-wheeled walker with self-locking mechanism made of gears and a spring (Approach 1), and a two-wheeled walker with a self-locking mechanism which uses a single spring (Approach 2). These combinations were tested in two quasi-experimental studies with pre–post test design. *Results*: No significant differences in duration, gait speed, and Expanded Timed Get Up and Go (ETGUG) were found between the walkers, but there was a marginally significant difference in Physiological Cost Index (PCIs), which means that the energetic cost with Approach 1 was greater than that with Approach 2. Users reported a feeling of insecurity and more weight, although no significant differences were observed and they were found to be equivalent in terms of safety. Study 2 found an improvement in duration and gait speed in the ETGUG between the different types of self-locking systems. *Conclusions*: The PCI is higher in the two-wheeled walker models and with the self-locking mechanism. Approach 2 did not show better conditions of use than the other two walkers, and participants did not highlight its braking system. Although safety is similar among the three walkers, further studies are needed, and the braking system of the two-wheeled walker needs to be improved (Approach 2).

## 1. Introduction

The population over 65 years of age seems to be growing fast all over the world [1], generating complex chronic situations which need to be solved. The normal ageing process is characterized by progressive body changes, especially at a functional level. In fact, one of the problems that most frequently affects the elderly population is the reduction of functional capacity [1] due to important impairments in the musculoskeletal system, with consequent mobility loss [2]. This natural process is the result of a complex interaction between the senescence of cells and their integrated functions. Environmental, physical, psychological, and social factors increase the risk of geriatric syndrome development, contributing also to the onset of geriatric comorbidities.

Changes associated with poor functional capacity and loss of independence are mainly related to physical and psychological decline. Joint function decline and a decrease in musculoskeletal capacity can contribute to the establishment of gait disorders, namely claudication, impaired gait pattern, and impaired ambulation [3]. Progressively, older adults tend to experience a gradual physical decline with loss of mobility, physical activity, strength, endurance, balance, and sensory functions. Besides, psychological decline leads to decrease in cognitive capacity and coping capacity, with mood changes. This type of physiological change contributes to a continuous perpetration of a sedentary lifestyle, since even the most simple and easy activities—walking, for example—are impaired. In fact, walking is a common and complex activity of daily living. It can be defined as the gait that humans use at low speeds [4]. Gait pattern can be strongly influenced by extrinsic and intrinsic factors [5], increasing with age from around 10% in those between 60 and 69 years of age to more than 60% in those aged over 80 years.

Some functions and systems are required to be intact for normal gait [5], like locomotor function, balance, postural reflexes, sensory function, motor control, and others. Aging is associated with the onset of many pathologies that negatively affect mobility and independence, causing deterioration in the systems contributing to the control of posture and balance [6]. Suspected or real threats to balance or other factors induce significant changes to the capacity to stand and walk [5].

It is estimated that around 30–40% of older people aged above 65 years can have gait changes, which can lead to falls [7]. Anticipatory postural adjustments also often fail, leading to imbalance, thus leading to falls. According to the National Program for Accident Prevention by the Portuguese Directorate-General for Health (DGS) [8], falls are the leading cause of injury among older people, representing a significant problem often leading to dependence and, consequently, to loss of quality of life. Therefore, there is a growing need among older people for assistive devices, which aim to improve mobility. According to Kaye and Kang [8], it is estimated that 6.1 million adults use assistive devices, of which 4.6% use a walker. In fact, older people often use walking aids to improve their ability to move around safely [6].

The necessity and the use of assistive devices increase with age, ranging from 14% to 18% in the healthy senior population to 45% to 96% in frail older adults [9]. These rates are expected to increase given the number of older people with multiple chronic conditions and mobility problems.

Currently, various technical aids that support walking are commercially available, including the so-called “walker” category. Walkers offer a wider and more stable base than other devices (canes and crutches). These devices are available in many sizes and are adjustable. Also, there are walkers with and without wheels, and special types of walkers [10]. They can be used as a reeducation tool, an intermediate phase before the use of another assistive device, or for permanent use. The purpose of these assistive devices is to reduce the weight load on the affected limbs, improve balance and/or reduce pain, and increase mobility and confidence during walking activities. People who use assistive devices report improved confidence and feelings of safety, leading to increased levels of activity and independence as well as possible physiological benefits of using these devices, namely improved cardiorespiratory fitness and prevention of osteoporosis [11]. Therefore, walkers should be selected based on the patients’ clinical criteria and degree of collaboration. Both safety and maintenance should be assessed, namely if the device’s height is adjusted and the existence of any loose rubber caps or hand grips [11].

There are fixed or articulated walkers with strategically placed fittings, with different structures and materials such as aluminum or light steel, with different slopes and forms of ground support, namely through two or four non-slip wheels. Some feature breaking systems with cables located on the handles; others have different accessories, from lanterns next to the handle, to baskets to transport personal objects; cushioned accent presentations are also possible features. Others show various adjustments and customizations, such as foldable structures and adjustable height, adjustable handles of independent regulation, and/or ergonomic handles. Nevertheless, no equipment has been found that integrates a self-locking wheel-drive system.

Many wheeled walkers have important limitations, such as the need to decrease gait speed when bypassing an obstacle or when there is the need to change direction [12]. The elderly person may not have enough strength or balance to control or stop the walker by him/herself. This is a negative outcome and needs to be improved. In addition to incorporated wheels, other innovations represent a significant progression on the efficacy and safety of current walkers, such as trunk support [13]. For gait rehabilitation [5], however, static walkers with no locking systems increase the risk of falling and the elderly might feel more insecure.

The use of walkers is a way to achieve independence and quality of life, for which factors like stability and balance are fundamental [14]. Nowadays, we can find robotic walkers (smart walkers), that represent a huge advance in assistive devices. Despite these advances, smart walkers often are developed through expensive and heavy materials [15]. Additionally, the current walkers are either too heavy, resulting in difficult gait and general motion, or are too complex to use, decreasing the chances of achieving optimal independence [14].

The most used walkers seem to be conventional walkers, which have the important feature of specific ground contact configuration [15]. The classic walker is usually a metal four-legged frame with rubber tips, and many do not present a built-in locking mechanism. There is a need to make the most used walkers more efficient, secure and usable.

The innovation presented here allows the walker to be braked whenever certain pressure is made on a component, avoiding the occurrence of falls due to forward imbalance by blocking the wheels, showing a competitive advantage for the safety of the user as compared to the available equipment on the market. Two different approaches for the self-locking systems were developed. This paper has the purpose of identifying the advantages and disadvantages specified by older adult users that experienced the self-locking systems through the performance of two quasi-experimental studies with a pre–post test design.

## 2. Development of Self-Locking Systems

The application of self-locking mechanisms for walkers is an innovative proposal, since this equipment is intended to aid people with mobility limitations due to weakness of lower limbs, which affects walking activities. In relation to current walkers with wheels, which slide as the progression of ambulation occurs due to the presence of front wheels, the innovative solution must guarantee that it is not necessary to raise the structure in order to walk. It also must allow, at any time, the locking of the walker whenever a certain sensation of imbalance is felt by the user. This imbalance is, in general, accompanied by an increase in pressure in the hand support of the walker. The new solutions must allow that patients with changes in the function of upper limbs and, at the same time, with changes in the mobility of lower limbs, are able to use a walker that integrates a self-locking system to help ambulation, strengthening safety measures.

### 2.1. Approach 1—System with Gears and a Spring

**Figure 1:**
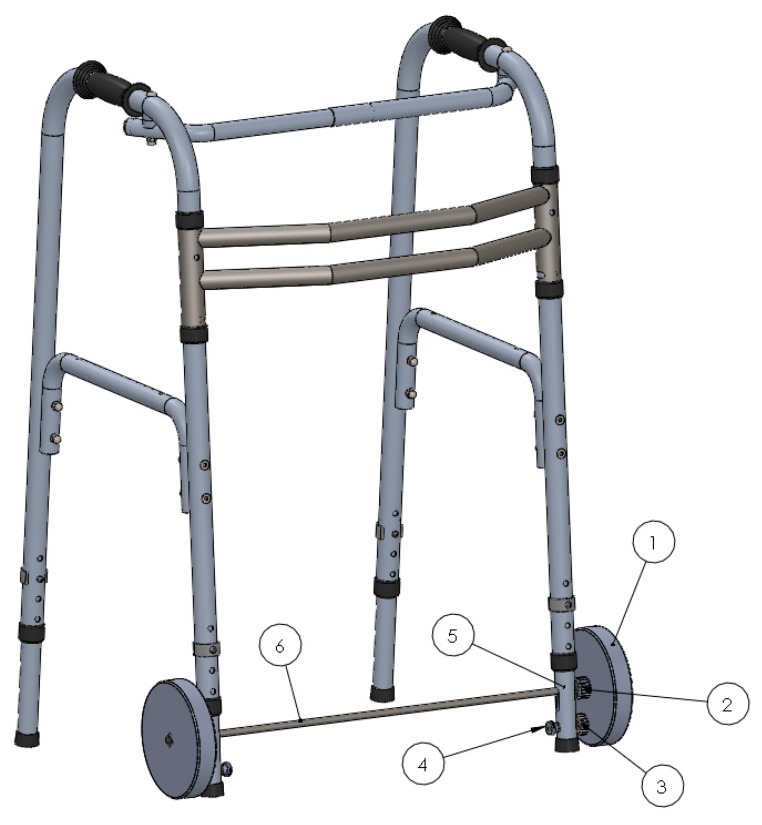
A three-dimensional Computer Aided Design (CAD) model of the walker set and the self-locking mechanism. 1—unidirectional wheels; 2—upper gear wheel; 3—the bottom gear wheel; 4—hand-operated nut; 5—walker’s front legs; 6—calibrated steel solid shaft (axis).

**Figure 2:**
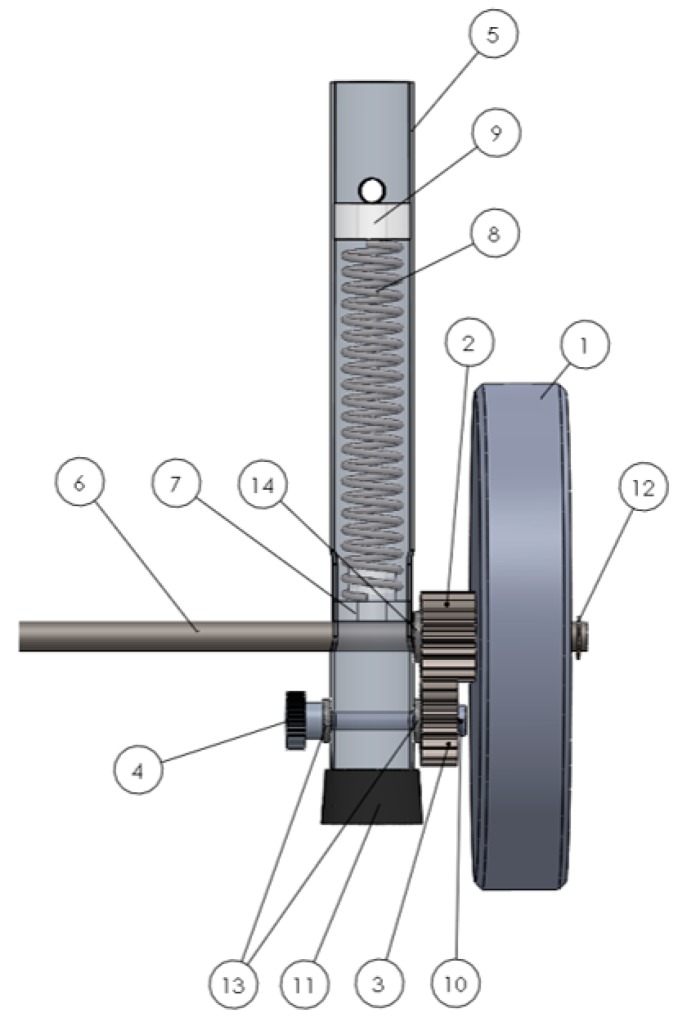
Details of the self-locking mechanism (Approach 1). 1—unidirectional wheels; 2—upper gear wheel; 3—the bottom gear wheel; 4—hand-operated nut; 5—walker’s front legs; 6—calibrated steel solid shaft (axis); 7—lock-guide; 8—helical compression springs; 9—stopper fixed inside the leg; 10—semi-screw; 11—rubber feet; 12, 13 & 14—(flat) washers.

**Figure 3:**
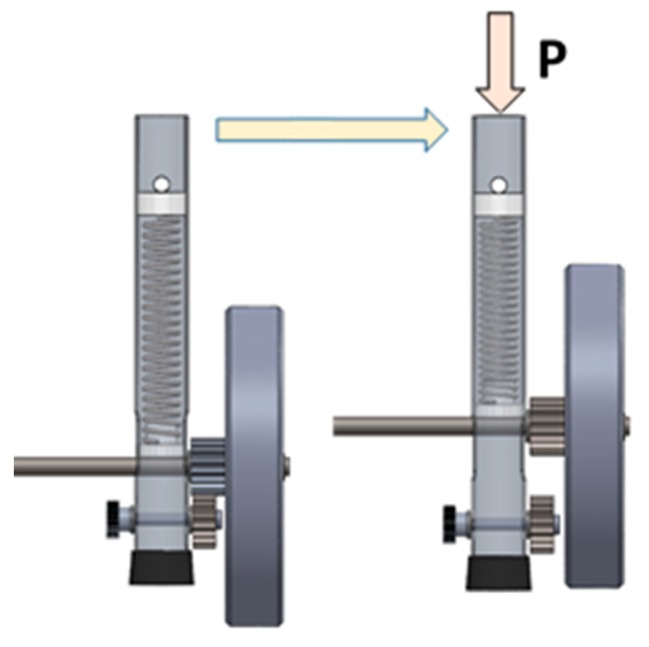
Functioning of self-locking mechanism (Approach 1).

This self-locking walker mechanism (Figure 1) consists of two unidirectional wheels (1) mounted on a calibrated steel solid shaft (6). The axis crosses the walker’s front legs (5) perpendicularly and crosses the semicircular holes, which allows for the upward–downward movement of the axis in the legs of the walker (5), as can be observed in Figure 1. Due to the gear presence, the calibrated steel solid shaft (6) is needed to ensure that both unidirectional wheels (1) have simultaneous rotation to avoid an offsetting in the rotational movement of the two wheels and to allow the rotation motion of the wheels around the axis (the relative motion between the couple wheel/axis). Figure 2 depicts the design details of the self-locking mechanism of Approach 1. The position of the unidirectional wheels (1) is maintained by the washers ((12) and (14)). When a certain pressure is applied to the “handles” of the walker, the resulting strength is transmitted to the helical compression springs (8), which are inside the legs of the walker (5). The springs (8) make contact with the stopper (9) fixed inside the leg and at the bottom with a lock-guide (7), exerting a force directly on the axis (6). The axis (6) imparts the force applied to the unidirectional wheels (1). If the pressure applied is sufficient, the springs (8) are compressed and the front legs (5) descend, until the rubber feet of the walker (11) make contact with the floor, as shown in Figure 3. This mechanism promotes greater stability to the user, when moving forward. The self-locking walker system further includes a mechanism that allows the wheels (1) to slide smoothly on a level surface by simply adjusting the knurled hand-operated nut (4). This nut (4) is screwed into a semi-screw (10), that works as an axis for the bottom gear wheel (3) and which is attached to the front legs (5) of the walker through the holes and the pair of flat washers (13). The nut (4), when tightened or loosened, causes a variation of the contact friction on the bottom gear wheel (3), which in turn, by means of engagement with the upper gear wheel (2) coupled to the walker’s wheel (1) by four screws, varies the ease with which the wheel (1) rotates about its axis. This mechanism eliminates the possibility that the walker may inadvertently roll on a sloping floor or, in other circumstances, where the walker’s unwitting movement must be prevented. The usability tests, as described in the following section, allowed the identification of some drawbacks based on the user’s feedback. However, from the technical point of view, some disadvantages can already be identified in the developed mechanism, such as the use of several heavy structural elements such as the gears, and if the transverse two-wheeled axis is applied to the rear legs of the walker, the use of walker will be unfeasible because the usability space cannot be accessed by the user. The use of Approach 1 is therefore limited to the front legs and inhibited by the wheels on the back of the walker. Approach 2 presents significant improvements in relation to Approach 1, for example by not incorporating a transverse shaft.

### 2.2. Approach 2—System with a Single Spring

**Figure 4:**
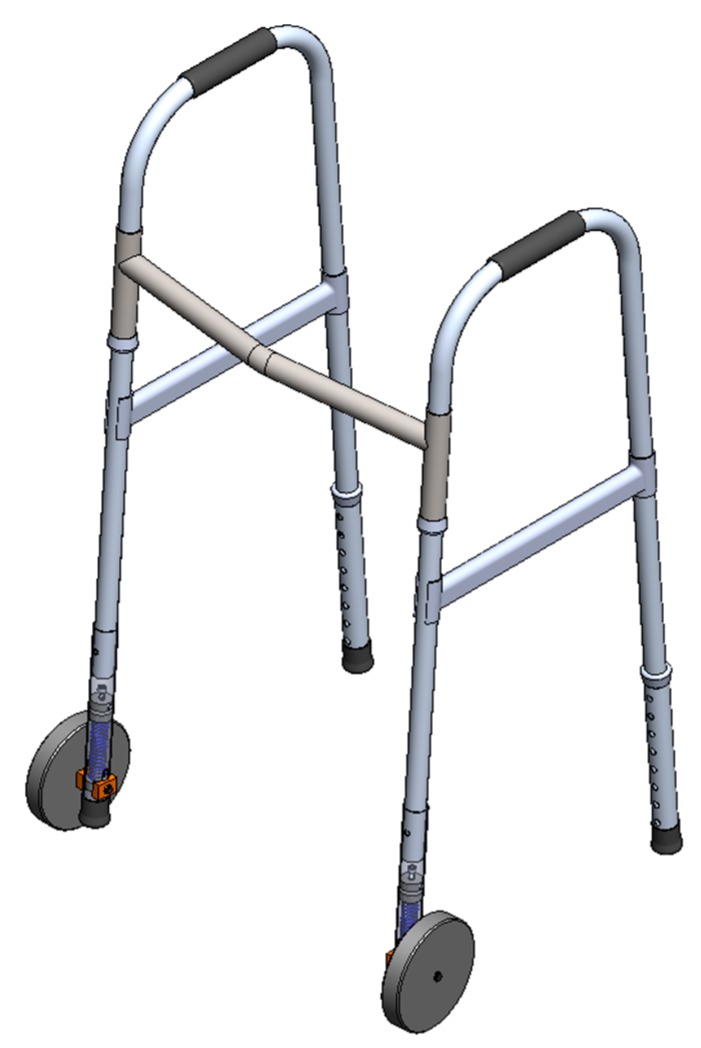
A three-dimensional CAD model of the walker set and the self-locking mechanism (Approach 2).

**Figure 5:**
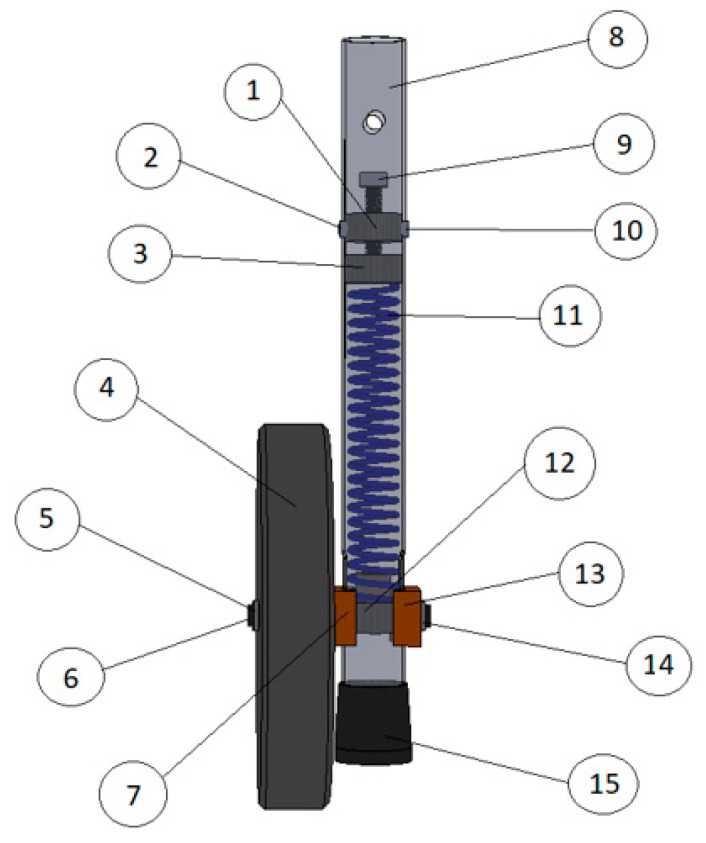
Details of the self-locking mechanism (Approach 2). 1—upper cylindrical wafer; 2 & 10—fixing bolts; 3—lower cylindrical wafer; 4—unidirectional wheel; 5—shaft; 6 & 14—washers; 7—interior sliding block; 8—walker leg; 9—feed screw; 10—semi-screw; 11—single helical compression spring; 12—stopper; 13—exterior sliding block; 15—rubber feet

**Figure 6:**
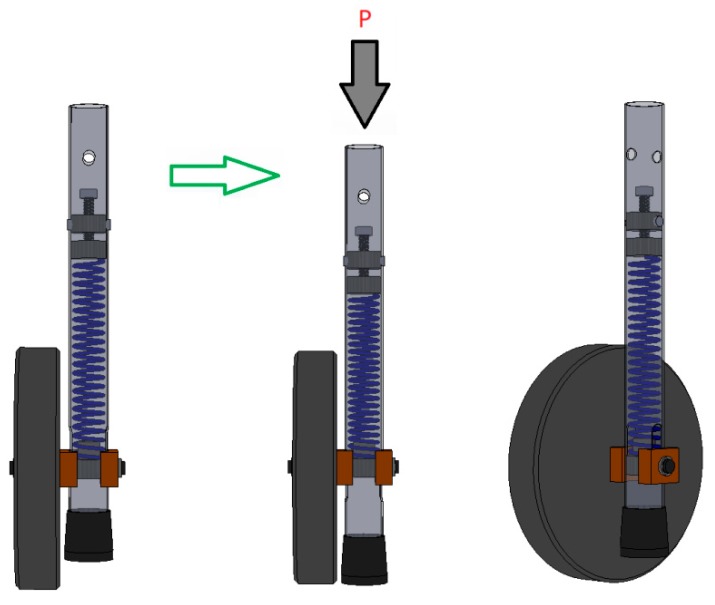
Functioning of self-locking mechanism (Approach 2).

The self-locking mechanism, shown in Figure 4, Figure 5, and Figure 6, is based on a system with a single helical compression spring (11) and one unidirectional wheel (4).

This new system can be easily assembled on each of the front legs of the walker. The system features are coupled on a new removable part with a diameter greater than that of the walker leg (8). The connection between the walker leg and the system is guaranteed through a locking pin at both ends. The unidirectional wheel is attached closer to the extremity of the removable part, through a shaft (5), and the side sliding is locked with two washers ((6) and (14)). Two sliding blocks, one interior (7) and other exterior (13), matching the part surface, ensure the correct position of the wheel, allowing the necessary axial sliding of the new feature. The compression spring (11) is blocked in the lower extremity with a stopper fixed inside (12). This stopper has a through hole with the same diameter of the shaft, which holds it in the proper position. In addition, it increases the contact surface of the shaft, reducing the cutting effect in the holes of the assembled part, where it crosses the shaft. The upper end of the stopper has a circular section with the inner diameter of the helical spring, working as a limiter. In the upper extremity, the compression spring is blocked in a spacer newly fixed with bolts ((2) and (10)). The spacer consists of two cylindrical wafers: a lower one (3), which makes contact with the end of the spring, and an upper one (1), with a central thread and a feed screw (9), with the end fixed on the lower wafer. This screw ensures the spacing of the two wafers, allowing the adjustment of the spring stiffness and thus the stiffness of the system. This means that the system can be adjusted to the user, namely to the force required to activate the spring. In fact, when a certain pressure is applied to the “handles” of the walker, the specific force is transmitted to the helical compression spring (11). If the applied pressure is sufficient, the spring compresses and the wheel assembly (wheel, shaft, and sliding blocks) slides vertically through the two lateral and longitudinal holes of the new system. As the wheel assembly moves vertically, the lower extremity of the system, where a rubber feet is placed (15), moves down and contacts with the floor, as shown in the Figure 6.

Just like Approach 1, this self-locking mechanism eliminates the possibility for the walker to inadvertently roll on a sloping floor or, in other circumstances, prevent the walker’s unwitting movement. However, the lightness and simplicity of these components makes the assembly lighter, eliminating the disadvantage identified in the first system. Furthermore, since the system can be applied independently on each wheel, it makes it more versatile and easy to carry, and may even be applied to the rear wheels, which was an impossibility on the Approach 1.

Both mechanisms, Approach 1 and Approach 2, allow mobility without having to raise the walker, in order to progress to ambulation. At the same time, they both prevent unwanted movement by automatic blocking of the system when certain pressure occurs. These devices protect the user from any unbalance, contributing to an increase in safety. Additionally, both systems cumulatively present a structure that is perfectly adjustable and adaptable, just like classic walkers, and easy to carry and sanitize. Nevertheless, the prototypes developed need clinical validation from a functional point of view. This topic is covered in Section 3.

## 3. Methods

The convenience sample was composed of older adults who used a walker and lived in nursing homes. Variables Expanded Times Up and Go (ETGUG) duration, gait speed, and energetic cost were assessed. From the users’ point of view, we established a comparison between the two solutions developed for the self-locking system: (1) Approach 1—using gears and a spring, and (2) Approach 2—using a single spring and the use of no self-locking system. Furthermore, advantages and disadvantages were identified for the solutions developed.

### 3.1. Study 1 Methods to Approach 1—System with Gears and a Spring

To test the walker with this system, a quasi-experimental study was developed (pre-post design, single group), using a non-probabilistic sample obtained by convenience (*n* = 18) of older users with a fixed walker and which were institutionalized in nursing homes. The data collection tool included: (1) Questions for sociodemographic characterization and clinical evaluation, based on the Satisfaction Assessment Scale in relation to a Technical Assistance (version 2.0), and (2) the ETGUG test, which included the action of walking with the classic fixed walker (FW) and the innovative two-wheeled walker with a self-locking mechanism (Approach 1, KW), for a total distance of 20 meters, in which the test was timed. There were measures for cardiac frequency and blood pressure before the test (at rest) and at the end of it. This data was used to calculate the speed (walking distance in meters × 60/time in seconds) and, subsequently, the Physiological Cost Index (PCI = (maximum heart rate per minute during the walking test—heart rate at rest per minute)/(speed)). The PCI was calculated for each course of the ETGUG test with each walker. All participants had a period of adaptation to the KW before the completion of the ETGUG. The users’ opinion regarding the innovative proposal of the two-wheeled walker with a self-locking mechanism (Approach 1, KW), was obtained through an open response questionnaire developed by the authors.

### 3.2. Study 2 Methods to Approach 2—System with a Single Spring

**Figure 7:**
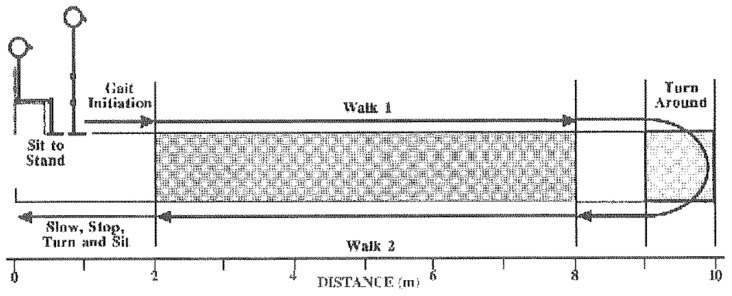
Expanded Timed Up and Go (ETGUG) test [13].

A second study was conducted through a quasi-experimental design (single group; pre–post test design). The convenience sample was composed of 40 older people living in nursing homes who used a walker. Among other variables, the participants had to perform the Expanded Timed Up and Go (ETGUG) test with the three types of walkers (fixed, two-wheeled with braking system (Approach 2), and four-wheeled walkers) to assess gait, speed, and fall risk. Satisfaction was also assessed with the previously mentioned questionnaire. According to Figure 7, the ETGUG allowed for the evaluation of (1) activity duration, (2) reverse walking speed for a total distance of 20 meters, and (3) energy expenditure using the PCI formula [12].

## 4. Results

### 4.1. Approach 1—System with Gears and a Spring

The participants’ perception regarding satisfaction of use suggests greater satisfaction with the FW in terms of weight (55.6%), comfort (66.6%), and effectiveness (66.6%) compared to the KW. Regarding ease of use, participants expressed more satisfaction with the KW (66.6%), as stability and security were considered more important for them (78.4%). As for personal opinion, respondents mentioned the following disadvantage: the KW was heavier, larger, and more insecure because it had wheels. On the other hand, the reported advantages were that they moved more quickly because they had a wheeled walker, which meant less strength exertion with the arms, and that the locking system was important, since it prevented falling events.

No statistically significant differences were found between duration (mean FW: 177.63; mean KW: 170.78; Z = −0.781; *p* = 0.435), gait speed (mean FW: 4.51; mean KW: 4.89; Z = −0.871; *p* = 0.384), and ETGUG for both walkers, but a marginally significant difference was found in PCIs (mean FW: 1.16; mean KW: 1.82; Z = −0.87; *p =* 0.068). This means that the energetic cost with KW was higher than that with FW.

### 4.2. Approach 2—System with a Single Spring

Preliminary results showed no differences in PCI for the three walkers (X^2^ = 2.177; *p* = 0.337) and in the final heart rate value (X^2^ = 0.770; *p* = 0.680). However, significant differences were found in duration (X^2^ = 15.65; *p* < 0.001) and gait speed (X^2^ = 15.80; *p* < 0.001) in the ETGUG between the three types of walkers. Regarding user satisfaction for the three walkers, significant differences were found in terms of stability and safety (X^2^ = 13.02; *p* < 0.001) and ease of use (X^2^ = 6.07; *p* < 0.005), as summarized in Table 1.

## 5. Discussion

Walking is one of the most recommended physical activities due to its simplicity, inexpensiveness, and accession [4]. Nevertheless, the capacity for physical performance, such as balance while walking or standing, decreases with age. Such problem can be solved using a walker—with or without wheels—improving balance and mobility for older persons [12]. Reduced ability to walk promotes the reduction of opportunities for individuals to participate in functional physical activities necessary to perform self-care, instrumental, and labor activities, leading to a greater loss of muscular strength and triggering a vicious circle of inactivity and weakness [2]. In this study, the physiological cost index (PCI) was used. The value of PCI is important, since the prescription of a walking technical aid depends on it. Ideal values can be obtained when the subject walks at a spontaneous comfortable speed [12].

There are several indications to prescribe a walking technical aid: some are related to the aging process, and others with neurological or osteoarticular pathologies [12]. Regarding the use of walkers, some authors [12] state that not all the users are satisfied with a wheeled walker. Usability and accessibility problems were identified as the main complaints, in agreement with the results of our study. In fact, the study presented here suggests that older people expend more physiological energy in walking using a two-wheeled walker with a self-locking mechanism (Approach 1, KW), than with classical fixed walker (FW), which is not in line with the results found by [12], who found higher PCIs with the fixed walker. The results of other studies and the consulted literature [15,16,17] confirm greater speed with the four-wheeled walker, which is thus a device of greater ease of use in relation to the two-wheeled walker (Approach 2). On the other hand, the two-wheeled walker (Approach 2) should allow a faster ETGUG than the fixed walker [18]. Stability and security should be valued with fixed walker, which is greater than that with a two-wheeled walker (Approach 2), since the braking system was not appreciated by participants. Additionally, some authors [12] indicate that one of the advantages of the fixed walker is its stability, though it requires adequate muscle and joint function in the upper limbs and preserved coordination and balance, which can be a problem since optimal limb function is normally absent. For this reason, a wheeled walker can be an adequate therapeutic solution, decreasing the possible load on the person that has to ambulate and progress without major issues. One study [15] noted a considerable number of issues regarding wheeled walkers triggering fall incidents. Based on their findings, the vast majority of older adults responded that it is challenging to maintain balance and open a door which is in the opposite direction of their walker [19,20,21,22].

This study reports that participants seem to be satisfied with FW in terms of weight, comfort, and effectiveness. They mentioned, as disadvantages, that KW was heavier, larger, and more unsafe for having wheels; however, they reported that the wheel-locking system was important for fall prevention. Therefore, KW needs improvement, namely in weight, dimension, comfort, effectiveness, and, possibly, its wheel-locking system. In summary, the two-wheeled walker (Approach 2) did not show better conditions of use in comparison to the other two walkers studied, but the wheel-locking system was highlighted by the participants. There is a need for further studies and the improvement of the wheel-locking system of the two-wheeled walker (Approach 2).

## 6. Conclusions

Currently, a variety of technical aids that support walking, such as walkers, are commercially available. There are fixed or articulated walkers with strategically placed fittings, with different structures and materials (such as aluminum or light steel), and with different slopes and forms of ground support, namely through two or four non-slip wheels. 

The equipment has a diverse range of characteristics, including different removable accessories like lights next to the handles, baskets for the transport of personal objects, or even cushioned seats. Some also feature various adjustable and customizable parts allowing for height adjustment, including handles of independent regulation and/or ergonomic handles.

Devices like walkers are intended to adapt to the needs of the population, optimizing use through less expenditure of energy with greater (positive) outcomes. The integration of a self-locking wheel-drive system is particularly important for preventing falls, which are a major issue in vulnerable populations like the elderly. In this paper, two different approaches to self-locking wheel-drive systems were presented, and the advantages and disadvantages were demonstrated for each. Both approaches allow walker immobilization after a certain pressure is exerted on the handle, avoiding falls due to forward imbalance. This represents a competitive advantage for the safety of the user with respect to the equipment that is currently on the market.

## Figures and Tables

**Table 1:** Differences in ETGUG duration by walker type.

Variables	Fixed Walker (*n* = 40) Friedman’s Mean Rank	Two-Wheeled Walker (Approach 2) (*n* = 40) Friedman’s Mean Rank	Four-Wheeled Walker (*n* = 40) Friedman’s Mean Rank	*X*^2^ (2)
PCI	2.03	2.15	1.83	2.177
FHR	1.91	1.99	2.10	0.770
ETGUG duration	1.93	2.43	1.60	15.80 *
Gait speed	2.15	1.50	2.35	15.80 *
Stability and safety	2.38	1.69	1.94	13.02 *
Ease of use	1.14	1.74	2.13	6.07 **

Friedman’s test: non-parametric statistical test to detect differences in treatments across multiple test attempts; X^2^: Wilcoxon’s test (non-parametric statistical hypothesis test to compare two related samples); PCI: Physiological Cost Index (maximum heart rate/minute during the walking test); FHR: frequency of heart rate (beats per minute, bpm); ETGUG: Expanded Timed Up and Go test (defined as the time spent in seconds to move 20 meters); Gait speed: expressed as the walking distance in meters, divided by 60 seconds; “Stability and safety” and “Ease of use” items: expressed as a Likert score from 1 to 5, with 1 being “dissatisfied” and 5 being “fully satisfied”. * *p* < 0.001, ** *p* < 0.005.

## References

[1] Wang T., Merlet J.-P., Sacco G., Robert P., Turpin J.-M., Teboul B., Guerin O. (2014). Walking analysis of young and elderly people by using an intelligent walker ANG. Robot. Auton. Syst..

[2] Marques-Vieira C.M.A., Mota de Sousa L.M., Carias J.F.M.M., Caldeira S.M.A. (2015). Nursing diagnosis “impaired walking” in elderly patients: integrative literature review. Rev. Gaúcha Enferm..

[3] Soeiro M.A.S. (2010). Envelhecimento Português. Desafios Contemporâneos—Políticas e Programas Sociais (Estudo de Caso). Master’s Thesis.

[4] Abadi F.H., Muhamad T.A., Salamuddin N. (2010). Energy expenditure through walking: Meta analysis on gender and age. Procedia Soc. Behav. Sci..

[5] Pirker W., Katzenschlager R. (2017). Gait disorders in adults and the elderly. Cent. Eur. J. Med..

[6] O’Hare M.P., Pryde S.J., Gracey J.H. (2013). A systematic review of the evidence for the provision of walking frames for older people. Phys. Ther. Rev..

[7] Tinetti M., Baker D., King M., Gottschalk M., Murphy T., Acampora D., Carlin B., Leo-Summers L., Allore H. (2008). Effect of dissemination of evidence in reducing injuries from falls. N. Engl. J. Med..

[8] Direção-Geral da Saúde [DGS] Programa Nacional de Prevenção de Acidentes 2010–2016. https://www.google.com/url?sa=t&rct=j&q=&esrc=s&source=web&cd=1&ved=2ahUKEwifpM-AlpjiAhXkxoUKHZRJCAwQFjAAegQIARAC&url=https%3A%2F%2Fwww.dgs.pt%2Fficheiros-de-upload-3%2Fdast-programa-nacional-de-prevencao-de-acidentes-pdf.aspx&usg=AOvVaw2UQdfUTEAB4Ep-738qUYVN.

[9] Demers L., Mortenson W., Fuhrer M., Jutai J., Plante M., Mah J., DeRuyter F. (2016). Effect of a tailored assistive technology intervention on older adults and their family caregiver: A pragmatic study protocol. BMC Geriatrics.

[10] Saad M., Greve J.M.D. (2007). Meios Auxiliares de Marcha. Tratado de Medicina de Reabilitação.

[11] Bradley S.M., Hernandez C.R. (2011). Geriatric Assistive Devices. Am. Fam. Physician.

[12] Lindemann U., Schwenk M., Klenk J., Kessler M., Weyrich M., Becker C. (2015). Problems of older persons using a wheeled walker. Aging Clin. Exp. Res..

[13] Poier P.H., Godke F., Foggiatto J.S., Ulbricht L. (2017). Development and evaluation of low-cost walker with trunk support for senior citizen. Rev. Esc. Enferm. USP.

[14] Martins M., Santos C., Frizera A., Ceres R. (2015). A review of the functionalities of smart walkers. Med. Eng. Phys..

[15] Martins M.M., Santos C.P., Neto A.F., Ceres R. (2012). Assistive mobility devices focusing on Smart Walkers: Classification and review. Rob. Auton. Syst..

[16] Braun T., Marks D., Zutter D., Grüneberg C. (2014). The impact of rollator loading on gait and fall risk in neurorehabilitation—A pilot study. Disabil. Rehabil. Assist. Technol..

[17] Teresa A.M.F. (2014). Estudo e Avaliação da adaptação andarilho-doença do utilizador. Master’s Thesis.

[18] Van Hook F.W.V., Demonbreun D., Weiss B.D. (2003). Ambulatory Devices for Chronic Gait Disorders in the Elderly. Am. Fam. Physician.

[19] Mansouri N., Goher K. (2016). Walking aids for older adults: review of end-user needs. Asian Soc. Sci..

[20] Cetin E., Muzembo J., Pardessus V., Puisieux F., Thevenon A. (2010). Impact of diferente types of walking aids on the physiological energy cost during gait for elderly individuals with several pathologies and dependent on a technical aid for walking. Ann. Phys. Rehabil. Med..

[21] Wall J.C., Bell C., Campbell S., Davis J. (2000). The Timed Getu-up-and-go Test Revisited: Measurement of the Component Tasks. J. Rehabil. Res. Dev..

[22] Edelstein J.E. (2013). Assistive Devices for Ambulation. Phys. Med. Rehabil. Clin..

